# A Novel Approach to Relocate Misplaced Proteins in Cells

**DOI:** 10.3390/biology14040420

**Published:** 2025-04-14

**Authors:** Grace Hohman, Ava Watson, Mohamed A. Eldeeb

**Affiliations:** Department of Chemistry, Illinois State University, Normal, IL 61761, USA

**Keywords:** protein quality control, protein localization, misfolded proteins, neurodegenerative disease, protein targeting

## Abstract

Proper cellular function hinges on appropriate subcellular protein localization. When cellular proteins become mislocalized, they can accumulate, cause cellular damage, and disrupt many biochemical and cellular processes. Notably, mislocalized protein accumulation and the resulting cytotoxic effects are salient features of neurodegenerative diseases including Alzheimer’s, Parkinson’s disease, and ALS. The detrimental cellular consequences of mislocalized proteins accumulation make it crucial to develop techniques and approaches that counteract this malfunction. Remarkably, a recent study by Ng et al. introduced targeted relocalization-activating molecules (TRAMs) as a novel molecular tool for relocalizing endogenous target proteins to counteract disease-associated mislocalized proteins. The authors developed a quantitative single-cell analysis to evaluate the strength and relocalization capability of TRAMs by coupling a target protein and a shuttle protein. Herein, we briefly highlight and discuss the potential molecular implications for targeted protein relocalization as an effective approach for correcting mislocalized proteins.

## Simple Summary

Humans are composed of millions of proteins that help regulate important cellular processes. However, these proteins must be in the correct location to function properly. Proteins that become mislocalized can accumulate and cause cell damage which is associated with various diseases. Thus, it is important to develop strategies to combat this improper localization in order to treat conditions associated with protein mislocalization and accumulation. Remarkably, a recent study conducted by Ng et al. identified a novel method to target mislocalized proteins and relocate them to their proper location. Through the quantitative analysis of single cells, the authors of this study demonstrated that targeted relocalization-activating molecules (TRAMs) could effectively relocate mislocalized proteins. This was accomplished by coupling the TRAMs with an additional protein, called a shuttle protein, to help facilitate relocalization. They showed that TRAMs coordinating with shuttle proteins could relocate mislocalized proteins as well as introduce protective functions in some cases. This new method of relocalization could offer a potential approach to counteract diseases associated with mislocalized proteins.

The proper localization of subcellular proteins is critical for cellular functioning and physiology. Thus, cells have evolved sophisticated quality control mechanisms to achieve proper localization of proteins, which is essential for maintaining cellular homeostasis. Proteins that are destined for specific organelles can be recognized through structural motifs or signal sequences. However, sometimes proteins can be recognized by the wrong targeting machinery due to similarities in signal sequences. This can prevent the proteins from reaching their proper cellular compartments. Additionally, mutations within the signal sequence of proteins can lead to mislocalization of these protein fragments, as seen in disease-related mutations of nuclear proteins associated with ALS. Furthermore, under some cell stress conditions, the import of proteins into the ER and mitochondria can be suppressed, leading to mislocalized proteins in the cytosol. Tellingly, the mislocalization of cellular proteins can lead to their accumulation, which can yield cytotoxic products and disrupt many biochemical and cellular processes. Of note, aberrant protein accumulation and the subsequent cellular damage are characteristic features of many neurodegenerative diseases including Alzheimer’s, Parkinson’s disease, and ALS [1,2].

Previous studies have demonstrated that mitochondrial import is inhibited in a number of neurodegenerative disease models, which could contribute to protein mislocalization. This is further supported by studies suggesting that increased protein mislocalization during aging may be due to the decline in effectiveness of mitochondrial protein import machinery and nuclear pore complexes. Interestingly, in neurodegenerative diseases, protein aggregates can also contribute to protein mislocalization by disrupting protein transport within different cellular compartments. Crucially, this protein mislocalization-mediated cellular dysregulation contributes to an increase in the abundance of mislocalized proteins in the cell ultimately interfering with normal cellular function and cooperatively drive cancer development and metastasis. Notably, mislocalized proteins have also been reported in several cancers. For example, the mislocalization of tumor suppressors or oncogenes have been associated with tumorigenesis [3].

The detrimental cellular consequences of mislocalized proteins make it crucial to develop techniques and approaches that address this malfunction. A recent study introduces targeted relocalization-activating molecules (TRAMs) as a molecular tool for inducing the relocalization of endogenous proteins to counteract disease-associated mislocalized protein fragments. The authors developed a quantitative single-cell analysis to evaluate the relocalization capability and strength of TRAMs by coupling a target protein and shuttle protein [4,5]. Herein, we briefly highlight the potential of targeted protein relocalization as an approach for correcting mislocalized proteins and their prospective translational implications.

Considering the deleterious consequences of abnormal proteins and their role in disease development and progression, a considerable amount of research has been investigating approaches to eliminate these proteins. One pivotal approach involves protein degraders, including proteolysis-targeting chimeras (PROTACs), to degrade target proteins. While this method has shown promising potential, it leads to the complete elimination of the target protein from the cell. Similarly, targeted autophagy inducers are another approach to degrade disease-associated protein aggregates by inducing the lysosomal degradation of these proteins [6]. While these methods offer novel strategies to target and eliminate cytotoxic proteins and protein aggregates, they do not specifically correct protein mislocalization, which can be the driving factor for cytotoxicity in some proteins. Remarkably, TRAMs offer a novel approach to correct the mislocalization of proteins without degrading proteins that have important functions in their proper cellular location.

**Figure 1:**
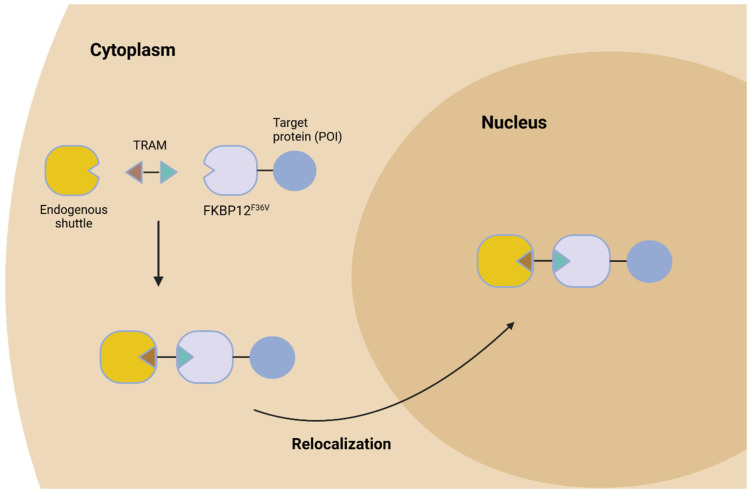
**Small molecules can elicit the targeted relocalization of proteins**. Bifunctional small molecules have been developed to bind to a protein called FKBP12F36V at one end and an endogenous or engineered shuttle protein in the cytoplasm or the nucleus at the other end. When FKBP12^F36V^ is fused to a target protein, the small molecules bind to the shuttle and the FKBP12^F36V^-tagged target protein simultaneously. The shuttle proteins subsequently transport the target protein between cellular compartments, for instance, from the cytoplasm into the nucleus. This method can be exploited to induce the targeted relocation of misplaced proteins inside mammalian cells.

What are TRAMs and how do they work? TRAMs are bifunctional small molecules designed to induce the relocalization of target proteins. Bifunctional small molecules bind to a target protein at one end, and an endogenous or engineered shuttle protein in the cytoplasm or the nucleus at the other end (Figure 1). Of note, Bifunctional molecules have diverse applications, including the simultaneous catalysis of two or more enzymatic processes and the dual inhibition of synergistic proteins involved in disease. While most classical therapeutic molecules modulate a single target, bifunctional molecules offer a stronger and more versatile approach when these single target drugs are not sufficient [7]. In the case of TRAMs, the shuttle protein that interacts with the TRAM contains strong localization sequences that help traffic the target protein to a specific location within the cell. To evaluate the TRAM-mediated specific relocalization of a target protein, the authors developed an approach to analyze two different proteins independently in the nucleus and the cytoplasm of a cell. Using this approach, they evaluated the TRAM-mediated relocalization of a nuclear-localized target protein, nicotinamide nucleotide adenylyltransferase 1 (NMNAT1), to the cytoplasm using a shuttle with a nuclear export sequence (NES). Their results demonstrated that the TRAM effectively mediated the relocalization of NMNAT1 from the nucleus to the cytoplasm in a dose-dependent manner. Importantly, the target protein was not degraded in any of the examined four cell lines treated with the TRAM. Next, the authors utilized the estrogen receptor (ERα) and glucocorticoid receptor (GR) as nuclear shuttle proteins and showed the successful nuclear enhancement of the target protein. Taken together, these results demonstrated the efficacy of harnessing TRAMs in the targeted localization of cellular proteins.

How does variation in shuttle protein concentration and strength affect TRAM-mediated relocalization? Experimentation with various shuttle protein concentrations revealed that an adequate amount of shuttle protein is necessary for TRAM-induced relocalization and varies depending on the nature of the shuttle protein and the target protein. The authors created a localization score to compare the localization strengths of different proteins which represents an important measurement for choosing a shuttle protein, depending on the desired levels of relocalization. Additionally, the authors observed different relocalization strengths between ERα and GR, which further underscores the importance of choosing an appropriate shuttle protein to induce the desired relocalization.

An inherent challenge for designing therapeutic approaches that target endogenous proteins for relocalization is the limited availability of small-molecule ligands for any given endogenous target protein. The authors overcame this challenge by installing binding domains on endogenous protein targets using a modified CRISPR)/CRISPR-associated protein 9 (cas9)-based gene editing technique. They then demonstrated that these endogenous proteins could be relocalized using other endogenous proteins as shuttles. Compared to ectopically expressed proteins, which only induced relocalization in a portion of the cell population in their models, endogenously expressed proteins used as shuttles for TRAMs affected the whole cell population

How can TRAMs be harnessed to modulate protein localization for therapeutic implementations? After demonstrating the efficacy of TRAM-induced relocalization, the authors aimed to test the relocalization of mutant proteins involved in diseases associated with mislocalized proteins. In neurodegenerative diseases, like Alzheimer’s, Parkinson’s disease, and ALS, mutations can contribute to the mislocalization of proteins prone to aggregation, which is a salient characteristic of these diseases [8,9,10,11]. Inducing the relocalization of mislocalized proteins could help prevent aggregate formation and potentially offer therapeutic benefits to patients with these conditions. To test this hypothesis, HeLa cells were designed to express one of three mutant proteins known for mislocalization in neurodegenerative diseases. Treatment with TRAMs resulted in the significant relocalization of each mutant protein. These results exemplify the potential role for TRAMs in mediating the relocalization of improperly localized proteins to prevent their proteotoxic effects, which are characteristic of many neurodegenerative diseases.

One of the proteins studied in these experiments was a mutated FUS protein, FUS^R495X^ which is observed in patients with ALS and leads to the formation of cytoplasmic granules in neurons. These granules are associated with the phenotype and pathogenic functions of ALS, making them an attractive therapeutic target [12]. The authors demonstrated the effective relocalization of mutated FUS proteins from the granules to the nucleus culminate in a reduction in the number of granules after treatment with TRAMs. These results supported the authors’ prediction that the TRAM-mediated removal and relocalization of mutant FUS proteins from granules could lead to the dissolution of FUS^R495X^ -positive cytoplasmic granules. Eliminating the cytoplasmic granules in neurons associated with pathogenic functions in ALS could potentially have therapeutic benefits. Additionally, the relocalization of mutant FUS proteins to the nucleus could help prevent the formation of granules. Further research is needed to determine the safety and efficacy of the TRAM-mediated relocalization of mutant FUS proteins as a potential therapy in the context of ALS physiological models.

Remarkably, the authors demonstrated the ability of TRAM-mediated relocalization to introduce gain-of-function activity in regions where target proteins were transported. Based on findings that a mutant protein involving mouse NMNAT1 (mNMNAT1), Wlds, has protective functions against Wallerian neurodegeneration [13], the authors tested if TRAM-mediated mNMNAT1 relocalization could have similar beneficial effects. The Wlds protein traffics to the axon where it exhibits protective functions after axonal damage [14]. To model this translocation, the authors engineered dorsal root ganglion (DRG) neurons to express mNMNAT1 linked to a shuttle protein. These cells were then treated with a TRAM designed to engage both the shuttle protein and an axon-localized protein. Their results showed the successful relocalization of mNMNAT1 to the axon. While nearly all control neurons lacking treatment with the TRAM died within 48 h after the cell body was removed, neurons treated with the TRAM exhibited extended axonal health for up to 96 h. These results suggest that mNMNAT1 had protective functions in the axon after relocalization. These findings expand the potential for TRAMs as a therapeutic tool by demonstrating their ability to mediate protein relocalization that introduces gain-of-function activity. Further research is warranted to determine the scope and nature of appropriate protein targets that could introduce beneficial gain-of-function activity when relocated to new cellular compartments.

## Concluding Remarks

While the recent study by Ng et al. introduced targeted relocalization-activating molecules (TRAMs) as a novel molecular tool for relocalizing endogenous target proteins to counteract disease-associated mislocalized proteins, a key limitation is that using GFP-tagged proteins to visualize relocalization may not applicable for endogenous protein targets in living cells. Therefore, further investigation into targeted protein relocalization approaches, in living cells using endogenous protein targets, is necessary to further enhance our understanding of TRAMs as a potential molecular tool for mediating the targeted relocalization of proteins. Despite this caveat, the results from Ng and colleagues unveil an elegant targeted protein relocalization approach and a promising potential for TRAMs as a novel technique to address mislocalized proteins that pose a threat to cellular health.

The mislocalization of aberrant proteins poses a challenge for cells that can have detrimental consequences on organismal health. Many neurodegenerative diseases are associated with aggregates of mislocalized proteins, which make them an attractive target for therapeutic agents. The new findings from Ng et al. demonstrate promising potential for the TRAM-mediated relocalization of target proteins as a tool for controlling the localization of mislocalized target proteins. The TRAM-mediated relocalization of target proteins to the nucleus from stress granules leads to a reduction in granules associated with neurodegenerative disease. Additionally, Ng and colleagues demonstrated protective, gain-of-function activity that could be introduced into new cellular compartments when TRAMs coordinate with shuttle proteins to mediate the relocalization of target proteins. These findings offer crucial insights into the molecular basis of TRAM-mediated relocalization as a potential therapeutic approach for neurodegenerative disorders and other conditions associated with mislocalized proteins. Further studies of TRAMs in physiological relevant cellular and protein models would extend our understanding of TRAMs and their translational implications.
